# Supporting the Sexual Health of People with Dementia: Developing Confident Care Teams Through Online Education

**DOI:** 10.1093/geroni/igaf122.2341

**Published:** 2025-12-31

**Authors:** Birgit Pianosi, Katelynn Aelick, Lori Schindel Martin, Kristy McKibbon, Rosemarie Mangiardi, Em Thielking

**Affiliations:** Laurentian University, Sudbury, Ontario, Canada; North Bay Regional Health Centre, Sudbury, Ontario, Canada; Toronto Metropolitan University, Toronto, Ontario, Canada; Hamilton Health Sciences, Hamilton, Ontario, Canada; Ontario Health, Toronto, Ontario, Canada; North Bay Regional Health Centre, North Bay, Ontario, Canada

## Abstract

Healthcare providers (HCPs) often feel unprepared to respond to the sexual health needs of people with dementia. Behavioural Supports Ontario’s (BSO) Sexual Expression and Dementia working group developed a 3-part e-learning series to address the need for HCP education in this domain. The program, Supporting the Sexual Health of People with Dementia, informed by expert consultation and over 300 academic sources, is designed to equip HCPs with the tools and resources they need to support people with dementia, specifically those residing in long-term care (LTC) homes. The first module of the program challenges common myths about sexuality and dementia. Module 2 provides practical tools and strategies for communicating with people living with dementia in LTC homes about their sexual health needs. The third module guides learners through assessing the risk of sexual expressions and outlines strategies to reduce risk in a manner that upholds LTC residents’ dignity. Throughout the series, learners engage in case studies of diverse people living with dementia. Learners follow LTC residents’ journeys as HCPs and care partners collaborate to understand and respond to residents’ unmet needs in person-centered ways. Following the launch of the online program in February 2025, it was evaluated using optional pre- and post-learning surveys. The purpose of the evaluation was to measure change in learners’ confidence and competence in supporting the sexual health of people with dementia. The results of this evaluation will be used to inform the development of future resources and education for healthcare providers on this topic.

